# Accelerating Global Interest in Genicular Artery Embolization: A Google Trends Analysis

**DOI:** 10.3390/jcm14227920

**Published:** 2025-11-08

**Authors:** Ann-Katrin Kaufmann-Bühler, Tazio Maleitzke, Alexander Hildebrandt, Tobias Winkler, Federico Collettini, Florian N. Fleckenstein

**Affiliations:** 1 Department of Radiology, Medical University of Graz, 8036 Graz, Austria; 2 Department of Diagnostic and Interventional Radiology, Corporate Member of Freie Universität Berlin and Humboldt-Universität zu Berlin, Charité—Universitätsmedizin Berlin, 10117 Berlin, Germany; 3 Center for Musculoskeletal Surgery, Corporate Member of Freie Universität Berlin and Humboldt-Universität zu Berlin, Charité—Universitätsmedizin Berlin, 10117 Berlin, Germany; 4 Julius Wolff Institute, Berlin Institute of Health, Charité—Universitätsmedizin Berlin, 10117 Berlin, Germany; 5 Trauma Orthopaedic Research Copenhagen Hvidovre (TORCH), Department of Orthopaedic Surgery, Copenhagen University Hospital—Amager and Hvidovre, 2650 Hvidovre, Denmark; 6 Department of Clinical Medicine, University of Copenhagen, 2200 Copenhagen, Denmark; 7 Berlin Institute of Health Center for Regenerative Therapies, Corporate Member of Freie Universität Berlin, Humboldt Universität zu Berlin and Berlin Institute of Health, Charité—Universitätsmedizin Berlin, 10117 Berlin, Germany; 8 BIH Charité Clinician Scientist Program, BIH Biomedical Innovation Academy, Berlin Institute of Health, Charité—Universitätsmedizin Berlin, 10117 Berlin, Germany

**Keywords:** arterial embolization, interventional radiology, osteoarthritis, knee, Google Trends

## Abstract

**Background/Objectives:** Genicular artery embolization (GAE) is an emerging, minimally invasive treatment for symptomatic osteoarthritis. This study assesses global online search trends for GAE compared with traditional knee replacement using Google Trends data. **Methods:** This retrospective observational study analyzed global search trends for osteoarthritis treatments using the publicly accessible Google Trends platform. Monthly relative search volumes (RSV; scaled 0–100) for English-language queries were retrieved from January 2018 to December 2024. Three emerging minimally invasive terms (“genicular artery embolization”, “knee embolization”, “interventional radiology”), and three traditional surgical terms (“knee arthroplasty”, “knee replacement”, “total knee replacement”) were included. Temporal trends were evaluated using linear and non-linear regression models, with model fit evaluated using coefficients of determination (R^2^), ANOVA, and accuracy measures. Statistical significance was defined as *p* < 0.05. **Results:** GAE-related terms demonstrated significant growth over time. “Genicular artery embolization” increased by 0.9% per month (R^2^ = 0.795, *p* < 0.001), and “knee embolization” increased at 0.9% per month (R^2^ = 0.627, *p* < 0.001) in a linear model. Traditional terms showed slower growth rates of 0.13–0.23% per month (R^2^ = 0.159–0.271). Exponential and quadratic models confirmed these patterns. Mean RSV values over the study period were higher for traditional procedures (e.g., “total knee replacement”: 83.13) than for GAE-related terms (e.g., “genicular artery embolization”: 22.5). **Conclusions:** Global online interest in GAE-related terms is increasing at a substantially faster rate than interest in traditional knee replacement. Rapidly growing search interest highlights the need for accurate and accessible online patient education regarding emerging treatments.

## 1. Introduction

Osteoarthritis (OA) is the most common joint disorder worldwide, currently affecting an estimated 24 million people [1,2]. Beyond the personal burden of chronic pain and restricted mobility, OA also has broader societal implications, such as reduced ability to work and decreased productivity [1].

Current clinical guidelines recommend non-surgical management as the first-line therapy for knee osteoarthritis. Recommended measures include patient education, structured exercise, physical therapy, weight management, non-steroidal anti-inflammatory drugs, and intra-articular corticosteroid injections [3,4]. When these approaches fail to adequately relieve symptoms and substantial pain or functional limitation persists, surgical intervention becomes the standard of care for advanced disease [5]. The predominant procedures are total knee arthroplasty (TKA) and unicompartmental arthroplasty, both of which aim to relief pain, restore joint function, and improve quality of life [6].

The number of joint replacement operations continues to rise worldwide, with an exponential increase expected over the coming years [2]. However, arthroplasty inherits perioperative risks and considerable healthcare costs. In medically complex patients, comorbidities and individual risk profiles may limit eligibility or delay surgical intervention.

A novel therapeutic approach to treating symptomatic OA is transarterial microembolization (TAME), first introduced by Okuno et al. TAME is a procedure within the field of interventional radiology (IR), involving the superselective embolization of synovial neovascularization, which arises from chronic inflammatory processes [7]. TAME provides effective pain relief and can be applied to various joints. When performed on the knee, the procedure is commonly referred to as genicular artery embolization (GAE). First long-term studies indicates that GAE leads to significant improvement in quality of life both shortly after treatment and in long-term follow-ups [8,9,10].

Over 5.7 billion people worldwide have internet access, making information on healthcare and medical treatments increasingly available [11]. Online data are commonly accessed resource for information on diseases, symptoms, treatments, and general health-related topics [12]. Individuals utilize a range of platforms, including support group websites, patient blogs, popular media outlets, and the webpages of medical organizations and health professionals [13]. Google Trends is an openly accessible data analysis tool that enables the quantification of public search interest for specific terms over time and according to geographical distribution [14]. This approach is increasingly utilized in medical research to provide insights into trends in public interest and the global distribution patterns of search behavior [15,16].

The purpose of this study is to compare global public search interest in two categories of OA treatment, (1) novel transarterial embolization technique and (2) traditional surgical procedures, using data extracted from Google Trends.

## 2. Materials and Methods

This retrospective observational study assessed global online search trends in GAE-related and traditional surgical treatment approaches for knee osteoarthritis using Google Trends data. English-language queries were conducted using six search terms related to knee OA treatment: three terms describing emerging minimally invasive techniques—“genicular artery embolization”, “knee embolization”, and “interventional radiology”—and three describing traditional surgical therapy—“knee arthroplasty”, “knee replacement”, “total knee replacement”. Each query was entered exactly as shown here under the “search term” option. The category filter was set to “All categories” and the geographic region to “Worldwide”. Monthly relative search volume (RSV) data were retrieved for the time period from January 2018 through December 2024. Data were downloaded as comma-separated values (CSV) files directly from the Google Trends interface. No additional normalization, smoothing, or transformation was applied prior to statistical analysis.

RSV values are scaled from 0 to 100, with a RSV of 100 denoting the highest level of search activity, while an RSV of 50 indicates that the search volume was half of the peak value. Google Trends does not provide exact search counts.

All queries and data retrieval were performed by a single researcher (A.K.) on 23 January 2025. A senior investigator supervised the process. The search terms were selected collaboratively by a clinical researcher and an expert in interventional radiology, focusing on relevance to both therapies and common usage in both professional and non-professional contexts. Abbreviations were excluded to avoid potential ambiguities.

### 2.1. Statistical Analysis

IBM SPSS Statistics version 29 (IBM Corp., Armonk, NY, USA) was used for statistical analysis. Monthly RSV data were analyzed using linear and non-linear regression models, including quadratic, cubic, logarithmic, and exponential growth functions. Model fit was assessed using coefficients of determination (R^2^), ANOVA, and accuracy measures such as mean absolute deviation (MAD), mean squared deviation (MSD), and mean absolute percentage error (MAPE). Level of significance was set at *p* < 0.05. Two analytical variants were tested to handle months with zero RSV. The first excluded initial zero RSV months before the first non-zero observation, while the second included all zeros as true values. Both model fits were evaluated using R^2^ and error metrics. Since early zero RSV months reflect periods before public awareness emerged, final results were reported using the model including all zero RSV values to preserve the natural temporal pattern of interest development.

### 2.2. Declaration of AI and AI-Assisted Technologies in the Writing Process

During the preparation of this paper, the authors used Large Language Models (ChatGPT-4o, OpenAI, San Francisco, CA, USA) for grammar and spelling correction of the manuscript. After using this tool, the authors reviewed and edited the content as needed and take full responsibility for the content of the publication.

## 3. Results

The temporal availability of search terms varied across the observation period. Between January 2018–December 2024, “genicular artery embolization” had 23 months with zero RSVs prior to its first non-zero occurrence, whereas “knee embolization” had 17 such months. No zero RSV values were observed for the traditional treatment terms or for “interventional radiology”.

**Figure 1:**
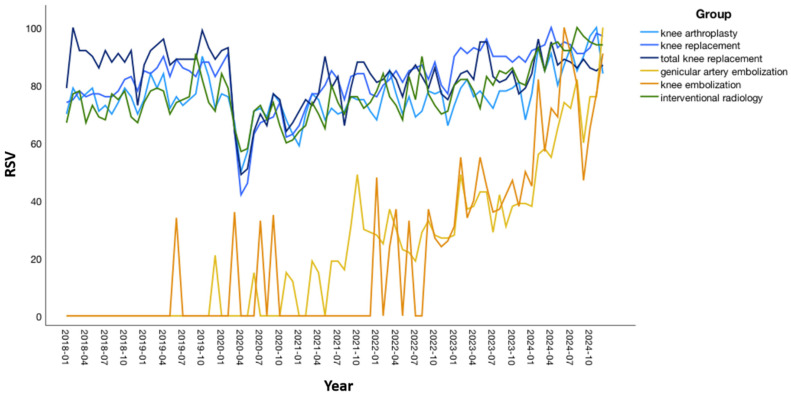
Monthly relative search volume (RSV) trends for six search terms related to knee osteoarthritis treatment (January 2018–December 2024). Traditional treatment terms and “interventional radiology” maintained consistently high RSVs, whereas “genicular artery embolization” and “knee embolization” showed a marked increase beginning in 2019–2020.

Descriptive analysis demonstrated consistently high mean RSVs for traditional treatment terms: “knee arthroplasty” (76.02 ± 8.12 SD), “knee replacement” (82.25 ± 10.54), and “total knee replacement” (83.13 ± 9.75), and lower mean RSVs for the emerging minimally invasive terms “genicular artery embolization” (22.45 ± 24.56) and “knee embolization” (21.19 ± 28.07). Table 1 summarizes monthly RSV distributions. Figure 1 depicts temporal trends in search interest across all six terms.

Linear regression models identified statistically significant monthly increases in RSV for “genicular artery embolization” (B = 0.90%, R^2^ = 0.795, MAPE = 24.45%) and “knee embolization” (B = 0.91%, R^2^ = 0.627, MAPE = 28.70%). More modest growth was observed for “knee arthroplasty” (B = 0.13%, R^2^ = 0.159, MAPE = 7.36%), “knee replacement” (B = 0.23%, R^2^ = 0.271, MAPE = 8.92%), and “interventional radiology” (B = 0.24%, R^2^ = 0.370, MAPE = 7.98%). No significant trend was detected for “total knee replacement” (B = −0.015, *p* = 0.741). Detailed performance metrics are provided in Table 2.

Quadratic regression improved overall model fit across all terms. Model improvement was determined based on higher R^2^ and lower error-based measures compared with linear regression. For “genicular artery embolization,” the model yielded R^2^ = 0.885 (B = 0.014, MAPE = 24.21%) and for “knee embolization” R^2^ = 0.787 (B = 0.021, MAPE = 29.91%). Traditional terms exhibited less curvature, with B = 0.007–0.008 (MAPE = 6.27–8.34%).

Growth models further supported these observations, with B of 0.221 and 0.203 for “genicular artery embolization” and “knee embolization,”, respectively (*p* < 0.001).

**Figure 2:**
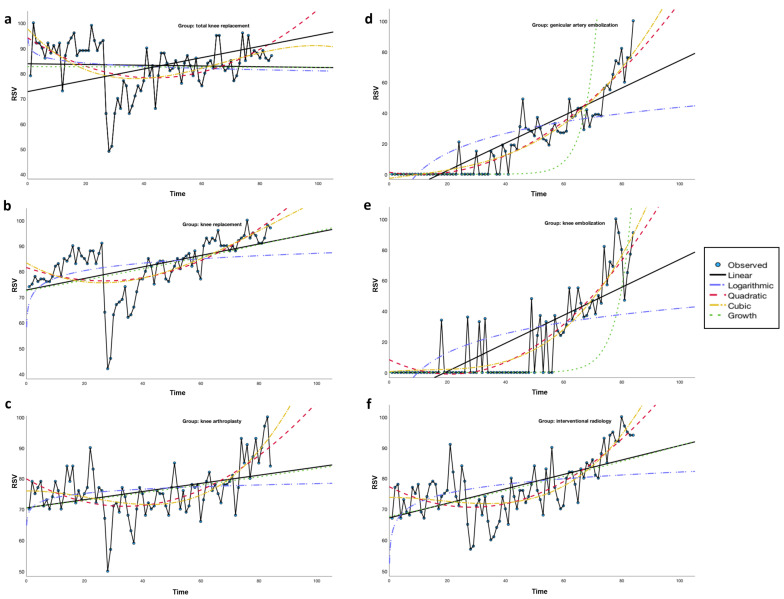
(**a**–**f**). Observed and modeled monthly relative search volume (RSV) trends for six knee osteoarthritis-related search terms (2018–2024). Panels show fitted curves for (**a**) “total knee replacement”, (**b**) “knee replacement”, (**c**) “knee arthroplasty”, (**d**) “genicular artery embolization”, (**e**) “knee embolization”, and (**f**) “interventional radiology”. Models include linear, logarithmic, quadratic, cubic and growth fits, illustrating steeper increase for minimally invasive terms compared to traditional surgical procedures.

Figure 2 illustrates the observed RSV trends, and Table 3 and Table 4 summarize corresponding model fits.

Including all zero RSV values in the model calculations yielded higher R^2^ for GAE-related terms across most model types (Table 5). Exceptionally large MAPE values for “genicular artery embolization” in the growth model were caused by divisions by small observed RSV values during early months, which disproportionately increase relative errors (Table 6).

## 4. Discussion

This study demonstrates a marked and statistically significant global increase in RSV for GAE-related terms. Compared with established surgical procedures, the steep rise in RSV for “genicular artery embolization” and “knee embolization” suggests growing public interest in minimally invasive options within IR.

Both linear and non-linear modeling confirmed that interest in this emerging procedure is expanding substantially faster than in traditional surgical procedures. Quadratic and exponential models exhibited the highest explanatory power, indicating that public interest is not only increasing but accelerating over time. In practical terms, this pattern suggests that recognition of GAE is growing exponentially, potentially reflecting the gain in visibility through early clinical adoption, research dissemination, and media exposure. By contrast, search interest in traditional surgical terms remained stable or showed only modest growth, and “total knee replacement” did not display a statistically significant trend, suggesting a plateau in public curiosity toward well-established treatments.

These findings align with prior research demonstrating heightened attention toward minimally invasive techniques in orthopedic care. For example, online search activity for intra-articular platelet-rich plasma injections in osteoarthritis has shown a steady increase [17]. Similar trends were reported for intra-articular hyaluronic acid injections among patients with knee osteoarthritis [18]. Within IR field, Berning et al. reported a growing interest in oncologic IR procedure in the United States, while global trends declined over the same period [19]. Comparable infodemiologic analyses have demonstrated that Google Trends data can capture public engagement with a range of medical topics, including cardiovascular disease and oncology [14,19,20].

Including the general term “interventional radiology” enabled contextual assessment of whether the observed increase in GAE-related searches reflected broader specialty awareness or a procedure-specific phenomenon. The stability of RSV for “interventional radiology” suggests that the increased attention is specific to GAE rather than a general expansion of interest in this field. This interpretation is consistent with findings by Dablan et al. (2025), who reported stable patterns for IR in the United States and Europe, but localized increase in Turkey [21].

Temporal variations in search activity likely reflect multifactorial influences, including seasonal interest, media coverage, and broader societal dynamics [22,23]. The pronounced decline in RSV across all terms during early 2020 coincides with the onset of the Coronavirus 2019 (COVID-19) pandemic, when public attention shifted toward infection control and elective procedures were widely deferred. Similar reductions in search interest for elective procedures, such as knee replacement, have been observed in other Google Trends analyses and mirror the documented decrease in elective procedures during this period [24,25,26].

Globally, online health-information seeking has become a major source of medical knowledge for patients [27,28,29]. A systematic review reported that approximately 55% of adult European adults searched for health-related information online in 2021—an increase of 21% compared with 2010. However, the quality of online information remains variable. Daraz et al. found that most publicly available healthcare information was of suboptimal quality, with none rated as excellent [30]. This disparity has implications for patient understanding and decision-making. In a cross-sectional study of patients undergoing nephrectomy, Chen et al. reported that individuals who searched for information online were significantly more likely to modify their treatment plans or raise additional questions during consultation [31]. Ichkawa et al. observed that online health-information seeking positively influenced shared decision-making among patients with systemic lupus erythematosus [32]. A nationwide Chinese survey further demonstrated that the quality of online information significantly affects treatment-related behaviors [33].

Current osteoarthritis management guidelines emphasize a patient-centered approach that integrates patients’ beliefs, expectations, and preferences into individualized treatment decisions [3]. Considering the growing prevalence of online information seeking, the rising public interest in GAE highlights the importance of ensuring that accurate, evidence-based digital content is available to the public. Providing credible online resources may help patients engage in informed, guideline-consistent decision-making and prevent misconceptions driven by incomplete or promotional content.

From a broader perspective, monitoring online search patterns can serve as early indicator of public awareness, information needs, and emerging demand for innovative therapies [14,34]. Integrating infodemiologic data into public-health surveillance and professional education strategies may support proactive dissemination of trustworthy information and help align public expectations with clinical practice.

This study has several limitations. The analysis was limited to English-language search terms and global Google Trends data, which may not capture regional variations or non-English search behavior. Google Trends provides relative, not absolute, search volumes and lacks demographic detail, precluding subgroup analyses. Only Google-based searches were included, potentially underrepresenting search queries conducted through other search engines or databases. This could lead to an incomplete picture of the overall online interest in the topics we examined. The presence of autocorrelation in the monthly RSV data may have influenced trend estimates, as consecutive observations are not statistically independent. Future analyses should apply time-series models that explicitly account for autocorrelation to improve precision. Finally, while increase search interest likely indicates heightened public awareness, it does not necessarily reflect increased clinical utilization or demand. Future studies linking search trends with procedural or registry data would be valuable to clarify the relationship between public awareness and real-world adoption of emerging therapies such as TAME.

## 5. Conclusions

This study demonstrates a strong rise in global search interest for GAE, suggesting growing public awareness of minimally invasive treatments for knee OA. In comparison, traditional surgical treatments showed relatively stable interest. While online trends do not directly reflect clinical demand, they emphasize the importance of providing accurate and accessible digital resources to support patient education. Future research should explore how search behavior influences treatment choices and healthcare utilization.

## Figures and Tables

**Table 1:** Summary statistics of monthly relative search volumes (RSV) for six knee osteoarthritis-related treatment terms (January 2018–December 2024).

		Group					
		Knee Arthroplasty	Knee Replacement	Total Knee Replacement	Genicular Artery Embolization	Knee Embolization	Interventional Radiology
**RSV**	Valid N	84	84	84	84	84	84
	Mean	76.0	82.3	83.1	22.5	21.2	77.1
	SD	8.1	10.5	9.8	24.6	28.1	9.4
	Median	76	84	85	19	0	76
	Prctl 25	71	77	79	0	0	70.5
	Prctl 75	79	90	89	37.5	37.5	82.5
	Min	50	42	49	0	0	57
	Max	100	100	100	100	100	100
	N preceding 0	0	0	0	23	17	0
	N total 0	0	0	0	35	48	0

PRCTL—Percentile; RSV—Relative search volumes; SD—Standard deviation.

**Table 2:** Linear regression analysis of monthly relative search volume (RSV) trends for six knee osteoarthritis-related search terms (January 2018–December 2024), including preceding zero values.

	Unstandardized Coefficients			Standardized Coefficients			
Group	B	95% CI of B LL	95% CI of B UL	Beta	95% CI of Beta LL	95% CI of Beta UL	*p*
knee arthroplasty	0.133	0.067	0.199	0.399	0.201	0.598	<0.001
knee replacement	0.225	0.145	0.305	0.521	0.336	0.705	<0.001
total knee replacement	−0.015	−0.101	0.072	−0.037	−0.253	0.18	0.741
genicular artery embolization	0.897	0.799	0.996	0.891	0.793	0.989	<0.001
knee embolization	0.911	0.759	1.063	0.792	0.659	0.924	<0.001
interventional radiology	0.235	0.169	0.302	0.609	0.437	0.78	<0.001

CI—Confidence Interval; LL—Lower Limit; UL—Upper Limit.

**Table 3:** Quadratic regression analysis of monthly relative search volumes (RSV) for six knee osteoarthritis-related search terms (January 2018–December 2024), including preceding zero values.

Group	Independent VAR	Unstandardized Coefficients			Standardized Coefficients			*p*
		B	95% CI of B LL	95% CI of B UL	Beta	95% CI of Beta LL	95% CI of Beta UL	
knee arthroplasty	Time	−0.527	−0.752	−0.303	−1.585	−2.258	−0.911	<0.001
	Time^2^	0.008	0.005	0.01	2.048	1.374	2.721	<0.001
knee replacement	Time	−0.389	−0.683	−0.095	−0.9	−1.581	−0.219	0.01
	Time^2^	0.007	0.004	0.011	1.466	0.785	2.147	<0.001
total knee replacement	Time	−0.735	−1.047	−0.422	−1.837	−2.619	−1.055	<0.001
	Time^2^	0.008	0.005	0.012	1.859	1.077	2.64	<0.001
genicular artery embolization	Time	−0.595	−1.576	0.386	−0.443	−1.174	0.288	0.24
	Time^2^	0.017	0.008	0.026	1.348	0.618	2.079	0
knee embolization	Time	−2.04	−3.084	−0.995	−1.367	−2.067	−0.667	<0.001
	Time^2^	0.032	0.022	0.042	2.196	1.496	2.896	<0.001
interventional radiology	Time	−0.471	−0.69	−0.253	−1.22	−1.785	−0.654	<0.001
	Time^2^	0.008	0.006	0.011	1.887	1.321	2.452	<0.001

CI—Confidence Interval; LL—Lower Limit; UL—Upper Limit; VAR—variable.

**Table 4:** Growth model regression analysis of monthly relative search volumes (RSV) for six knee osteoarthritis-related search terms (January 2018–December 2024), including preceding zero values.

Group	Unstandardized Coefficients			Standardized Coefficients			*p*
	B	95% CI of B LL	95% CI of B UL	Beta	95% CI of Beta LL	95% CI of Beta UL	
knee arthroplasty	0.00167	0.00078	0.00256	0.376	0.176	0.577	<0.001
knee replacement	0.00278	0.00163	0.00392	0.465	0.274	0.657	<0.001
total knee replacement	−0.00004	−0.00118	0.00111	−0.007	−0.223	0.21	0.951
genicular artery embolization	0.197	0.143	0.251	0.683	0.496	0.869	<0.001
knee embolization	0.241	0.184	0.298	0.718	0.548	0.887	<0.001
interventional radiology	0.00294	0.00207	0.00381	0.589	0.415	0.764	<0.001

CI—Confidence Interval; LL—Lower Limit; UL—Upper Limit.

**Table 5:** Comparison of coefficients of determination (R^2^) for linear and non-linear trend models of relative search volumes (RSV) for six knee osteoarthritis-related search terms (January 2018–December 2024), with and without preceding zero values.

Coefficients of Determination R^2^ (Excluding Preceding Zeros)											
Group	Excluded Cases ^†^	Linear	95% CI (LL; UL)	Logarithmic	95% CI (LL; UL)	Quadratic	95% CI (LL; UL)	Cubic	95% CI (LL; UL)	Growth Model	95% CI (LL; UL)
Knee arthroplasty	0	0.159	(0.041; 0.319)	0.05	(0.000; 0.175)	0.416	(0.249; 0.570)	0.445	(0.279; 0.595)	0.142	(0.031; 0.299)
Knee replacement	0	0.271	(0.119; 0.437)	0.135	(0.028; 0.291)	0.402	(0.236; 0.558)	0.406	(0.240; 0.561)	0.217	(0.078; 0.382)
Total knee replacement	0	0.001	(0.000 *; 0.062)	0.033	(0.000 *; 0.145)	0.212	(0.075; 0.378)	0.229	(0.087; 0.395)	0	(0.000 *; 0.049)
Genicular artery embolization	23	0.794	(0.680; 0.872)	0.727	(0.585; 0.827)	0.832	(0.735; 0.896)	0.853	(0.767; 0.910)	0.466	(0.271; 0.636)
Knee embolization	17	0.637	(0.477; 0.759)	0.508	(0.325; 0.662)	0.772	(0.655; 0.853)	0.774	(0.658; 0.855)	0.515	(0.333; 0.667)
Interventional radiology	0	0.37	(0.205; 0.530)	0.177	(0.052; 0.340)	0.588	(0.437; 0.710)	0.605	(0.457; 0.723)	0.347	(0.184; 0.509)
**Coefficients of Determination R^2^ (Including Preceding Zeros)**											
**Group**	**Excluded Cases ^†^**	**Linear**	**95% CI (LL; UL)**	**Logarithmic**	**95% CI (LL; UL)**	**Quadratic**	**95% CI (LL; UL)**	**Cubic**	**95% CI** **(LL; UL)**	**Growth model**	**95% CI (LL; UL)**
Knee arthroplasty	0	0.159	(0.041; 0.319)	0.05	(0.000; 0.175)	0.416	(0.249; 0.570)	0.445	(0.279; 0.595)	0.142	(0.031; 0.299)
Knee replacement	0	0.271	(0.119; 0.437)	0.135	(0.028; 0.291)	0.402	(0.236; 0.558)	0.406	(0.240; 0.561)	0.217	(0.078; 0.382)
Total knee replacement	0	0.001	(0.000 *; 0.062)	0.033	(0.000 *; 0.145)	0.212	(0.075; 0.378)	0.229	(0.087; 0.395)	0	(0.000 *; 0.049)
Genicular artery embolization	0	0.795	(0.700; 0.862)	0.486	(0.322; 0.629)	0.885	(0.827; 0.924)	0.888	(0.832; 0.926)	0.724	(0.606; 0.812)
Knee embolization	0	0.627	(0.483; 0.740)	0.349	(0.186; 0.511)	0.787	(0.690; 0.856)	0.796	(0.703; 0.863)	0.583	(0.431; 0.706)
Interventional radiology	0	0.37	(0.205; 0.530)	0.177	(0.052; 0.340)	0.588	(0.437; 0.710)	0.605	(0.457; 0.723)	0.347	(0.184; 0.509)

^†^ Excluded cases represent months with zero RSV values prior to the first non-zero occurrence. * The lower limit of the 95% CI for R^2^ is set to 0 when the calculation yields a negative value (0 ≤ R^2^ ≤ 1). CI—Confidence Interval; LL—Lower Limit; UL—Upper Limit.

**Table 6:** Comparison of model fit metrics (MAD, MSD, MAPE) for linear and non-linear regression models of monthly relative search volumes (RSV) for six knee osteoarthritis-related search terms (January 2018–December 2024) including preceding zero values.

	MAD				
Group	o_lin	o_log	o_squ	o_cub	o_gr
Knee arthroplasty	5.43	5.64	4.62	4.42	5.37
Knee replacement	6.36	7.10	5.83	5.82	6.49
Total knee replacement	7.33	7.13	6.35	6.29	7.49
Genicular artery embolization	8.72	13.31	5.69	5.88	90.51
Knee embolization	13.70	18.35	8.75	8.60	15.46
Interventional radiology	5.99	6.82	4.64	4.61	5.91
	**MSD**				
**Group**	**o_lin**	**o_log**	**o_squ**	**o_cub**	**o_gr**
Knee arthroplasty	54.74	61.84	38.06	36.11	54.45
Knee replacement	80.05	94.94	65.64	65.22	79.19
Total knee replacement	93.87	90.90	74.03	72.49	94.36
Genicular artery embolization	122.43	306.17	68.76	66.77	72346
Knee embolization	290.56	506.28	166.13	158.50	607.87
Interventional radiology	55.31	72.24	36.21	34.70	54.00
	**MAPE**				
**Group**	**o_lin**	**o_log**	**o_squ**	**o_cub**	**o_gr**
Knee arthroplasty	7.36%	7.61%	6.27%	6.05%	7.23%
Knee replacement	8.92%	9.87%	8.08%	8.06%	9.01%
Total knee replacement	9.67%	9.45%	8.34%	8.22%	9.78%
Genicular artery embolization	24.45%	30.39%	24.21%	22.65%	249.8%
Knee embolization	28.70%	31.60%	29.91%	29.21%	80.04%
Interventional radiology	7.98%	9.03%	6.26%	6.21%	7.83%

MAD—mean absolute deviation; MAPE—mean absolute percentage error; MSD—mean squared deviation; o_cub—cubic model; o_gr—growth model; o-lin—linear model; o_log—logarithmic model; o_squ—quadratic model.

## Data Availability

The datasets used and/or analyzed during the current study are available from the corresponding author upon reasonable request.

## Abbreviations

CI	Confidence Interval
COVID-19	Coronavirus disease 2019
CSV	Comma-separated values
GAE	Genicular artery embolization
IR	Interventional radiology
LL	Lower Limit
MAD	Mean absolute deviation
MAPE	Mean absolute percentage error
MSD	Mean squared deviation
OA	Osteoarthritis
PRCTL	Percentile
RSV	Relative search volumes
SD	Standard deviation
TAME	Transarterial microembolization
TKA	Total knee arthroplasty
UL	Upper Limit
VAR	Variable
